# Development and external validation of an artificial intelligence model for predicting mortality and prolonged ICU stay in postoperative critically ill patients: a retrospective study

**DOI:** 10.1186/s13017-025-00650-2

**Published:** 2025-10-15

**Authors:** Dong Jin Park, Seung Min Baik, Kyung Sook Hong, Heejung Yi, Jae Gil Lee, Jae-Myeong Lee

**Affiliations:** 1 Department of Laboratory Medicine, Eunpyeong St. Mary’s Hospital, College of Medicine, The Catholic University of Korea, Seoul, Korea; 2 Division of Critical Care Medicine, Department of Surgery, Ewha Womans University Mokdong Hospital, Ewha Womans University College of Medicine, Seoul, Korea; 3 Department of Surgery, Korea University College of Medicine, Seoul, Korea; 4 Division of Critical Care Medicine, Department of Surgery, Ewha Womans University Seoul Hospital, Ewha Womans University College of Medicine, Seoul, Korea; 5 Division of Acute Care Surgery, Department of Surgery, Korea University Anam Hospital, Korea University College of Medicine, Goryeodae-ro 73, Seongbuk-gu, Seoul, 02841 Republic of Korea

**Keywords:** Artificial intelligence, Mortality, Length of stay, Risk factors, Postoperative complications, Ensemble model

## Abstract

**Background:**

Existing predictive models in critical care, specifically for postoperative critically ill patients, often struggle to accurately predict prolonged intensive care unit (ICU) stays, a key aspect of patient care. The integration of artificial intelligence (AI) offers a promising approach for bridging this gap. We aimed to develop an AI-based model to predict mortality and prolonged ICU stay in postoperative critically ill patients, enhance prognostic accuracy, and address the shortcomings of current models.

**Methods:**

This retrospective study included data from 6,029 postoperative critically ill patients from two medical centers, including a wide range of clinical, surgical, and laboratory variables. Multiple machine-learning models, including extreme gradient boosting, light gradient boosting, category boosting, random forest, and multilayer perceptron, were employed. A soft-voting ensemble model was developed to aggregate the strengths of individual models. The models underwent external validation, and the SHapley Additive exPlanations (SHAP) method was utilized to assess the impact of various features on predictions.

**Results:**

In internal validation, the ensemble model demonstrated superior performance with an area under the receiver operating characteristic curve (AUROC) of 0.8812 for mortality and 0.7944 for prolonged ICU stay. It achieved 0.9095 accuracy and an F1 score of 0.7014 for mortality predictions. For prolonged ICU stay, it attained an accuracy of 0.9368 and an F1 score of 0.5762. During external validation, the model maintained high performance, with an AUROC of 0.8330 for mortality and 0.7376 for prolonged ICU stay. It showed 0.9200 accuracy and an F1 score of 0.6768 for mortality and 0.9028 accuracy with an F1 score of 0.5689 for prolonged ICU stay. SHAP analysis confirmed that key predictors, including emergency surgery, serum osmolality, lactate levels, and diastolic blood pressure, remained significant.

**Conclusions:**

This study represents a significant advancement in the application of AI in critical care, especially for postoperative critically ill patients. The developed AI model outperformed existing models in predicting mortality and prolonged ICU stay, demonstrating notable accuracy and reliability. Its ability to identify critical, under-emphasized clinical factors could enhance decision-making in critical care settings. Although promising, further validation in diverse clinical settings is essential to confirm the model’s efficacy and broader applicability.

**Supplementary Information:**

The online version contains supplementary material available at 10.1186/s13017-025-00650-2.

## Background

Early and accurate prognosis is crucial in critical care medicine, where rapid decisions regarding life and death are often required. The integration of advanced technologies, particularly artificial intelligence (AI), has revolutionized this field. The ability of AI to process and interpret large datasets is increasingly being applied to critical care prognosis and management, representing a significant advancement in the discipline [1, 2].

Critically ill patients can be broadly classified into two categories: those with an internal medicine background who experience an acute episode of a chronic disease or a sudden emergency, and surgical patients whose treatment trajectory typically begins with surgical intervention. Surgical patients differ significantly from internal medicine patients, whose conditions often develop gradually. Postoperative critically ill patients, in particular, face unique challenges and complications that necessitate predictive models tailored to their specific pathophysiological profile and risk factors [3, 4]. Developing a high-performance mortality prediction model would provide surgeons with additional insights beyond clinical information, enabling them to focus more effectively on their postoperative patients.

An important gap in current predictive models is their limited focus on prolonged intensive care unit (ICU) stay, especially for postoperative patients. Despite advances in critical care, mortality rates for postoperative patients remain high, ranging from 10 to 50% [3–5]. Additionally, predicting prolonged ICU stays is an equally critical but underexplored area in existing models. The ability to predict such outcomes is not only clinically relevant but also has significant operational implications. Prolonged ICU stays are often associated with more complex clinical needs, and effective predictive models could help optimize ICU resource allocation and management [6, 7].

Several mortality prediction models have been established, including the Acute Physiology and Chronic Health Evaluation (APACHE), Simplified Acute Physiology Score (SAPS), and Sequential Organ Failure Assessment (SOFA). However, these models primarily focus on the general population of critically ill patients. Although they are informative, their accuracy in predicting mortality varies, generally ranging from moderate to high. For instance, APACHE and SAPS scores typically achieve an accuracy of 70–80%, depending on the patient cohort and clinical setting. However, these models fail to account for the unique characteristics of postoperative critically ill patients. This specific patient group presents a unique combination of factors, such as surgical stress, the interaction of preexisting comorbidities, and acute physiological changes induced by surgery, which are not adequately captured by existing models [8–10].

We aimed to develop a model capable of accurately predicting mortality and prolonged ICU stay in critically ill patients following surgery. Furthermore, the developed AI model was used to analyze the key variables influencing mortality and prolonged ICU stay. By doing so, we sought to identify factors that are currently underestimated in clinical practice. The development process involved applying advanced machine-learning techniques to extensive datasets from multiple centers, ensuring a comprehensive and externally validated approach.

## Methods

### Patient selection and data collection

We conducted a retrospective study of 6029 critically ill postoperative patients, with 3478 patients selected from Center A and 2551 from Center B. Comprehensive data on clinical, surgical, and laboratory variables were collected for each patient. Specifically, clinical data included sex, age, body mass index (BMI), APACHE II score, and medical history. The surgical data included the nature of the surgery (routine or emergency), duration of the operation, and surgical department. Laboratory data encompassed vital signs and the results of 67 blood tests. The primary outcomes, defined according to the study objectives, were mortality and ICU length of stay (LOS), with LOS categorized as either longer or shorter than 30 days. This threshold was chosen for two reasons: (i) 30-day outcomes are widely recognized as a meaningful endpoint in surgical and critical care research, with 30-day mortality being a common benchmark; and (ii) in the Korean healthcare system, ICU stays exceeding 30 days are formally classified as long-term admissions, reflecting substantial clinical and resource burdens. Although shorter cutoffs (7 or 14 days) are often reported in the literature, our focus on the 30-day threshold aligns with international outcome standards and local clinical practice.

The total number of data points recorded at Center A was 306,064, with 49,018 (16.0%) missing data points. At Center B, 224,488 data points were recorded, with 48,194 (21.5%) missing data points.

### Data partitioning and model validation

**Fig. 1:**
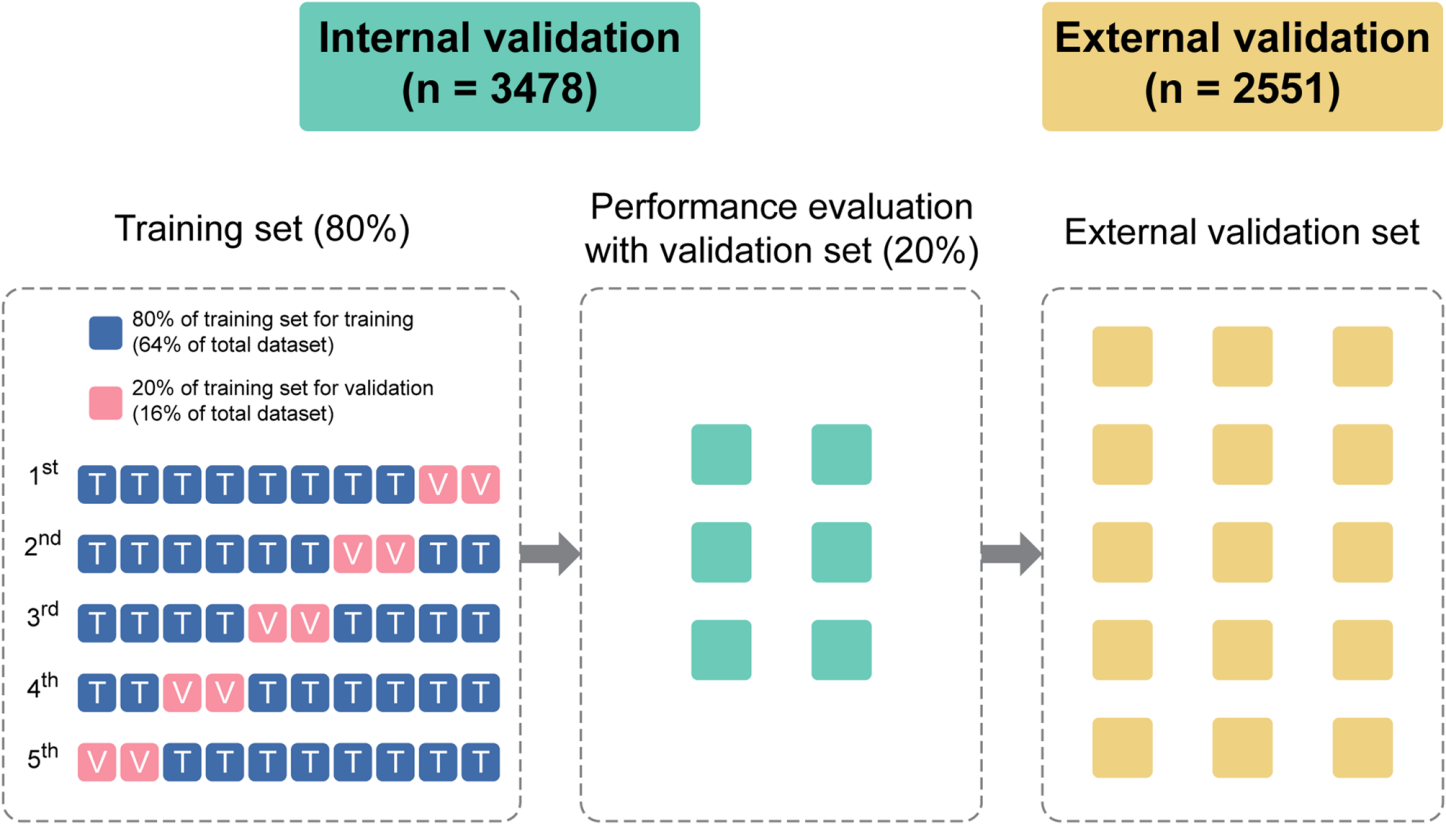
Data partitioning, model validation, and stratified K-fold validation of the study. Hierarchical segmentation of data from Center A (*n* = 3478), with 80% allocated for training and 20% for testing. The training set was further split into nested training and validation subsets in an 8:2 ratio. Stratified K-fold validation was used to ensure robust and representative sample division

We implemented a hierarchical data segmentation strategy on the dataset obtained from Center A. Initially, 80% of the patients were allocated to the training set, and the remaining 20% were reserved for the test set (Fig. 1).

The training set was further segmented to ensure proper model calibration and mitigate overfitting. Specifically, it was split at an 8:2 ratio to create nested training and validation subsets. This nested segmentation was implemented using stratified K-fold validation with five splits (n_splits = 5) (Fig. 1).

Layered K-fold validation is an advanced cross-validation technique that enhances the standard K-fold method. In traditional K-fold validation, the data are randomly split into “K” subsets. The model is trained “K” times, using “K-1” folds for training and the remaining single fold for validation. This process is repeated until each fold has served as the validation set. Stratification adds an extra layer of sophistication by ensuring that each fold is representative of the entire dataset. For example, in mortality prediction, the relative proportions of each class (e.g., surviving vs. non-surviving) are maintained across all folds. This approach is particularly advantageous for medical datasets, in which class imbalances are common. By avoiding scenarios where a fold lacks representation of certain classes, stratified K-fold validation ensures a more robust and reliable validation process.

### Model selection and description

We selected the following models to develop our predictive framework. eXtreme Gradient Boosting (XGBoost), which uses the gradient boosting algorithm, is an ensemble learning method that sequentially adds predictors to minimize the loss function. By focusing on correcting errors in previous predictors, this algorithm is particularly effective at improving model performance and handling complex datasets [11]. The Light Gradient Boosting Machine (LGBM) is a tree-based algorithm that utilizes the gradient boosting framework. It employs a histogram-based approach to partition data, reducing memory usage and accelerating training. Known for its efficiency, LGBM is well-suited for handling large datasets and tasks requiring fast training and high performance [12]. Categorical Boosting (CatBoost) is a boosting algorithm that is well-suited for handling categorical features directly. Incorporating categorical information during training minimizes the need for extensive data preprocessing, such as one-hot encoding. CatBoost optimizes an ensemble of decision trees focused on categorical data, making it highly effective for tasks involving these features [13]. Random Forest (RF) is an ensemble machine-learning method that builds a “forest” of decision trees during training. Using a bagging technique, it trains multiple decision trees on subsets of data through bootstrapping. For classification tasks, RF predicts the mode of class outputs from individual trees, and for regression tasks, it computes the average prediction. RF leverages the diversity of trees to improve accuracy and handle complex data patterns [14]. A multilayer perceptron (MLP) is a type of artificial neural network comprising interconnected nodes organized into three or more layers: an input layer, one or more hidden layers, and an output layer. The network learns by adjusting the weights and biases of connections between nodes through a process called backpropagation. MLPs are particularly adept at capturing complex data patterns and are widely used for tasks such as image recognition, natural language processing, and regression [15].

The handling of missing data depends on the model. The three boosting models—XGBoost, LGBM, and CatBoost—can handle missing values without imputation. In contrast, for the RF and MLP models, missing data points were imputed using the median. All modeling processes were implemented using the scikit-learn library.

### Ensemble modeling

To enhance the predictive power of the individual models, we developed an ensemble model using a soft-voting technique. The development was as follows: Each model in the ensemble independently made predictions for the task at hand. For example, in a binary classification problem, each model produced a probability score between 0 and 1, representing the likelihood of belonging to one of two classes. In soft voting, the predictions of each model were weighted based on their estimated confidence or performance. Models with higher accuracy or better performance for similar data were assigned greater weights, whereas less confident models received lesser weights. The weighted predictions from all models were combined to create a final ensemble forecast. This was achieved by calculating the weighted average of the prediction probabilities. For instance, in the binary classification scenario, the final prediction was the weighted average of the probability scores. Ensemble models operate on the principle of blending the results of different models to improve overall accuracy and reduce overfitting. By integrating diverse models, ensemble techniques capture a broader range of data patterns and complexities, often yielding superior predictive performance [16].

### Modeling and external validation

After initial modeling and calibration using the dataset from Center A, the developed model was applied to the dataset from Center B, which served as the external validation cohort. This step evaluated the model’s generalizability and performance across different patient populations and clinical settings.

### Parameter impact analysis using SHapley additive explanations

To gain insight into the factors influencing the model’s predictive performance, we employed the SHapley Additive exPlanations (SHAP) method. SHAP values provide a consistent measure of feature importance and their impact on model predictions. The SHAP method is applicable to various machine-learning models, including the MLP, a deep-learning model used in this study. SHAP quantifies the magnitude and direction of each variable’s impact on the model output, providing transparent and interpretable insights into the inner workings of AI models [17–19].

## Results

### Demographic and clinical characteristics of patients

The demographic and clinical characteristics of the enrolled patients are presented in Table 1. We compared the two centers, Center A (n = 3478) and Center B (n = 2551), analyzing various patient variables in the survival and death groups after surgery. At Center A, the proportion of men in the death group (n = 316) was 62.7%, which was higher than that in the survival group (n = 3162) (*P* = 0.002). The mean age was 66.8 years in the survivor group and 69.8 years in the decedent group (*P* < 0.001). The mean BMI was 23.4 kg/m^2^ in survivors and 22.8 kg/m^2^ in decedents (*P* = 0.036). The APACHE II score was significantly higher in decedents (31.1 points) than in survivors (21.6 points) (*P* < 0.001). Emergency surgery was performed in more decedents (64.2%) than survivors (36.7%) (*P* < 0.001). The operative time was shorter in decedents (129.6 min) than in survivors (152.0 min) (*P* = 0.001), and the length of ICU stay was longer in decedents (14.4 days) than in survivors (5.6 days) (*P* < 0.001). Regarding medical history, there were significant differences in diabetes (*P* = 0.004) and kidney disease (*P* = 0.003), but no differences were observed in other conditions, such as drinking history (*P* = 0.205) or smoking history (*P* = 0.966). At Center B, 50.3% of the survivors (n = 2371) were men, compared to 62.0% of the decedents (n = 178) (*P* = 0.002). The mean age and BMI were similar between the two groups (*P* = 0.405 and *P* = 0.027, respectively). As in Center A, the APACHE II score was higher in decedents (*P* < 0.001). Emergency surgery was more common in decedents (44.7%) than in survivors (19.4%) (*P* < 0.001). The operative time was slightly longer in survivors (168.9 min) than in decedents (149.0 min) (*P* = 0.038), and the ICU LOS was longer in decedents (16.6 days) than in survivors (6.1 days) (*P* < 0.001). The smoking history in Center B was significantly different from that in Center A (*P* = 0.013), whereas other medical histories were similar between the two groups. Differences were also observed in the specialties; neurosurgery (*P* = 0.006) and orthopedics (*P* = 0.004) had a higher percentage of deceased patients. Vital signs showed a higher pulse rate in deceased patients at both centers (*P* < 0.001), whereas other parameters, such as blood pressure, respiratory rate, and body temperature, showed slight or no differences. In addition to clinical information, we collected 58 laboratory results. The detailed results are shown in Additional file 1.

**Table 1:** Demographic and clinical characteristics of patients from two medical centers

Variables	Center A (n = 3478)			Center B (n = 2551)		
	Survival group (n = 3162)	Deceased group (n = 316)	*P*-value	Survival group (n = 2371)	Deceased group (n = 178)	*P*-value
Sex, male (%)	1698 (53.7)	198 (62.7)	0.002	1193 (50.3)	111 (62.0)	0.002
Age (years)	66.8 ± 15.9	69.8 ± 14.3	< 0.001	67.7 ± 14.8	68.6 ± 15.5	0.405
BMI (kg/m^2^)	23.4 ± 3.8	22.8 ± 3.9	0.036	23.7 ± 4.0	23.0 ± 3.9	0.027
APACHE II score	21.6 ± 8.5	31.1 ± 9.2	< 0.001	22.0 ± 8.2	31.8 ± 10.2	< 0.001
Emergency surgery (%)	1160 (36.7)	203 (64.2)	< 0.001	461 (19.4)	80 (44.7)	< 0.001
Operation time (min)	152.0 ± 119.7	129.6 ± 107.7	0.001	168.9 ± 122.9	149.0 ± 129.9	0.038
Period of ICU stay (days)	5.6 ± 11.2	14.4 ± 18.7	< 0.001	6.1 ± 12.4	16.6 ± 20.9	< 0.001
*Past medical history* (%)						
Alcohol consumption history	864 (27.4)	76 (24.1)	0.205	511 (21.6)	42 (23.5)	0.552
Smoking history	726 (23.0)	73 (23.1)	0.966	454 (19.2)	48 (26.8)	0.013
Hypertension	1595 (50.4)	164 (51.9)	0.622	1285 (54.2)	98 (54.7)	0.882
Diabetes mellitus	830 (26.2)	107 (33.9)	0.004	682 (28.8)	51 (28.5)	0.941
Cardiovascular	366 (11.6)	43 (13.6)	0.285	253 (10.7)	18 (10.1)	0.798
Respiratory	38 (1.2)	5 (1.6)	0.559	24 (1.0)	2 (1.1)	0.892
Neurology	57 (1.8)	6 (1.9)	0.903	64 (2.7)	5 (2.8)	0.940
Kidney	60 (1.9)	14 (4.4)	0.003	71 (3.0)	6 (3.4)	0.787
Liver	109 (3.4)	14 (4.4)	0.367	64 (2.7)	6 (3.4)	0.606
Malignancy	378 (12.0)	44 (13.9)	0.307	233 (9.8)	17 (9.5)	0.888
Department (%)						
Surgery	1295 (41.0)	125 (39.6)	0.630	925 (39.0)	76 (42.5)	0.360
Thoracic surgery	264 (8.3)	29 (9.2)	0.613	303 (12.8)	18 (10.1)	0.290
Oral and maxillofacial surgery	27 (0.9)	1 (0.3)	0.308	10 (0.4)	0 (0)	0.384
Urology	110 (3.5)	8 (2.5)	0.375	91 (3.8)	2 (1.1)	0.061
Neurosurgery	753 (23.8)	100 (31.6)	0.002	731 (30.8)	73 (40.8)	0.006
Orthopedics	560 (17.7)	45 (14.2)	0.121	253 (10.7)	7 (3.9)	0.004
Plastic surgery	36 (1.1)	1 (0.3)	0.174	23 (1.0)	1 (0.6)	0.583
Obstetrics and gynecology	44 (1.4)	1 (0.3)	0.107	29 (1.2)	2 (1.1)	0.901
Otorhinolaryngology	65 (2.1)	5 (1.6)	0.568	6 (0.3)	0 (0)	0.501
Ophthalmology	8 (0.3)	1 (0.3)	0.832	1 (0.04)	0 (0)	0.784
*Vital signs*						
Systolic blood pressure (mm Hg)	124 ± 21	122 ± 23	0.184	127 ± 20	124 ± 24	0.086
Diastolic blood pressure (mm Hg)	73 ± 13	71 ± 14	0.095	73 ± 12	71 ± 15	0.125
Pulse rate (bpm)	82 ± 17	93 ± 23	< 0.001	78 ± 17	90 ± 19	< 0.001
Respiratory rate (bpm)	18 ± 5	19 ± 6	0.337	18 ± 4	19 ± 7	0.043
Body temperature (℃)	36.8 ± 1.7	36.3 ± 3.7	0.015	36.8 ± 0.6	36.7 ± 1.0	0.197

BMI, Body mass index; APACHE, Acute Physiology and Chronic Health Evaluation; ICU, Intensive care unit

### Comparative analysis and external validation of mortality prediction performance of machine learning, deep learning, and ensemble models

**Fig. 2:**
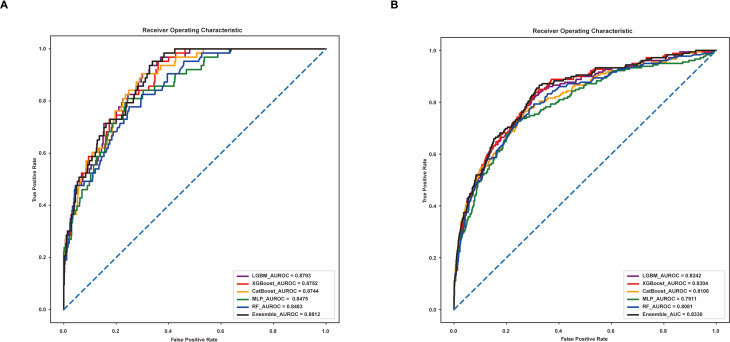
Area Under the receiver operating characteristic curve for mortality predictions of the models. **A** AUROC of the initial model. **B** AUROC of the externally validated model. AUROC, area under the receiver operating characteristic curve

In the initial development of mortality prediction models (Table 2; Fig. 2A), the LGBM model, with a cutoff of 0.20, demonstrated the best performance among individual models, recording an area under the receiver operating characteristic curve (AUROC) of 0.8793, accuracy of 0.9138, precision of 0.7368, recall of 0.6667, and an F1 score of 0.6937. The XGBoost model, also with a cutoff of 0.20, closely followed, achieving an AUROC of 0.8752, accuracy of 0.9066, precision of 0.7139, recall of 0.6985, and an F1 score of 0.7058. The CatBoost model, at a 0.22 cutoff, had an AUROC of 0.8744, accuracy of 0.9059, precision of 0.7186, recall of 0.6572, and an F1 score of 0.6811. The RF model, with a 0.25 cutoff, had an AUROC of 0.8483, accuracy of 0.9009, precision of 0.7011, recall of 0.7097, and an F1 score of 0.7052. The MLP model, with a 0.30 cutoff, achieved an AUROC of 0.8475, accuracy of 0.8980, precision of 0.6838, recall of 0.6652, and an F1 score of 0.6738. The ensemble model, which combined predictions using a soft-voting technique with varying weights of 0.6 for LGBM, 0.15 each for XGBoost and CatBoost, and 0.05 each for RF and MLP, delivered the best overall performance. It achieved an AUROC of 0.8812, accuracy of 0.9095, precision of 0.7213, recall of 0.6858, and an F1 score of 0.7014.

**Table 2:** Comparative evaluation of model efficacy in mortality prediction for postoperative critically ill patients

Model (cutoff)	AUROC	Accuracy	Precision	Recall	F1 score
XGBoost (0.20)	0.8752	0.9066	0.7139	0.6985	0.7058
CatBoost (0.22)	0.8744	0.9059	0.7186	0.6572	0.6811
LGBM (0.20)	0.8793	0.9138	0.7368	0.6667	0.6937
Random Forest (0.25)	0.8483	0.9009	0.7011	0.7097	0.7052
MLP (0.30)	0.8475	0.8980	0.6838	0.6652	0.6738
Ensemble model^*^ (0.20)	0.8812	0.9095	0.7213	0.6858	0.7014

XGBoost, eXtreme Gradient Boosting; CatBoost, Categorical Boosting; LGBM, Light Gradient Boosting Machine; MLP, multilayer perceptron

^*^Using a soft-voting technique, the five models were assigned different weights: 0.6 for LGBM, 0.15 for XGBoost and CatBoost, and 0.05 for Random Forest and MLP

The external validation results, presented in Table 3; Fig. 2B, further supported the robustness of the ensemble model. Using data from Center B, the ensemble model again outperformed individual models, with an AUROC of 0.8330, accuracy of 0.9200, precision of 0.6873, recall of 0.6678, and an F1 score of 0.6768. Among the individual models, XGBoost demonstrated the highest performance, with an AUROC of 0.8304, accuracy of 0.9083, precision of 0.6599, recall of 0.6795, and an F1 score of 0.6689. The LGBM model had an AUROC of 0.8242, accuracy of 0.9192, precision of 0.6785, recall of 0.6467, and an F1 score of 0.6605. CatBoost achieved an AUROC of 0.8106, accuracy of 0.9287, precision of 0.7167, recall of 0.6311, and an F1 score of 0.6607. The RF model had an AUROC of 0.8081, accuracy of 0.9012, precision of 0.6467, recall of 0.6809, and an F1 score of 0.6613. The MLP model, with the lowest performance among the models, exhibited an AUROC of 0.7911, accuracy of 0.9071, precision of 0.6435, recall of 0.6427, and an F1 score of 0.6431. The consistent superior performance of the ensemble model across the internal development and external validation datasets highlights its robustness, generalizability, and efficacy in mortality prediction.

**Table 3:** External validation of AI models for mortality prediction in postoperative critically ill patients

Model (cutoff)	AUROC	Accuracy	Precision	Recall	F1 score
XGBoost (0.20)	0.8304	0.9083	0.6599	0.6795	0.6689
CatBoost (0.22)	0.8106	0.9287	0.7167	0.6311	0.6607
LGBM (0.20)	0.8242	0.9192	0.6785	0.6467	0.6605
Random Forest (0.25)	0.8081	0.9012	0.6467	0.6809	0.6613
MLP (0.30)	0.7911	0.9071	0.6435	0.6427	0.6431
Ensemble model* (0.20)	0.8330	0.9200	0.6873	0.6678	0.6768

XGBoost, eXtreme Gradient Boosting; CatBoost, Categorical Boosting; LGBM, Light Gradient Boosting Machine; MLP, multilayer perceptron

^*^Using a soft-voting technique, the five models were assigned different weights: 0.6 for LGBM, 0.15 for XGBoost and CatBoost, and 0.05 for Random Forest and MLP

### Comparative analysis and external validation of prolonged ICU stay (30 days) prediction performance of machine-learning, deep-learning, and ensemble models

**Fig. 3:**
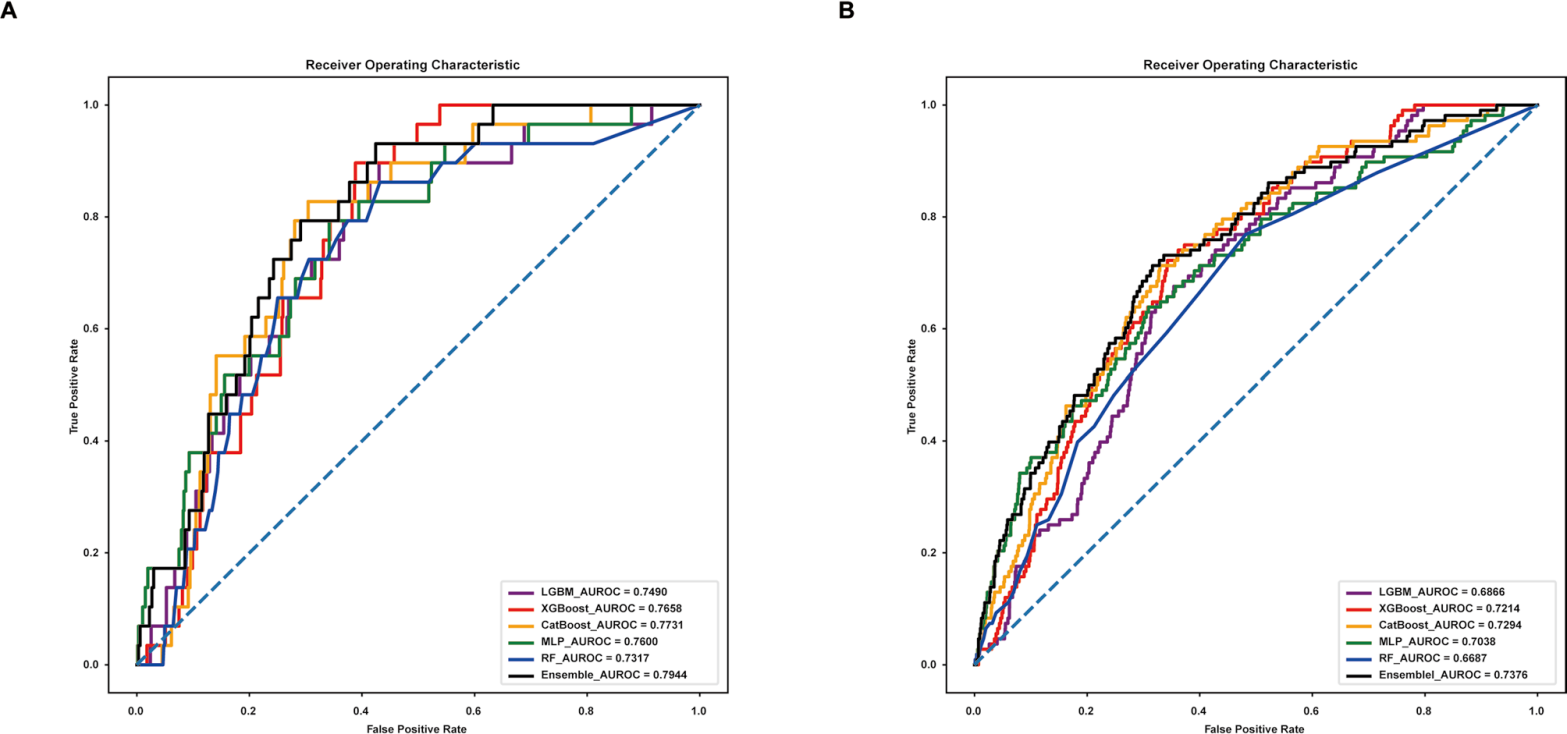
Area under the receiver operating characteristic curve for prolonged ICU stay predictions of the models. **A** AUROC of the initial model. **B** AUROC of the externally validated model. AUROC, area under the receiver operating characteristic curve

Table 4; Fig. 3A summarize the predictive performance for an ICU stay longer or shorter than 30 days. The MLP model, with a cutoff of 0.11, showed strong performance, achieving an AUROC of 0.7600, accuracy of 0.8879, precision of 0.5573, recall of 0.6282, and an F1 score of 0.5719. The CatBoost model, at a 0.07 cutoff, had an AUROC of 0.7731, accuracy of 0.8506, precision of 0.5553, recall of 0.6747, and an F1 score of 0.5648. The XGBoost model, with a cutoff of 0.07, achieved an AUROC of 0.7658, accuracy of 0.8376, precision of 0.5366, recall of 0.6184, and an F1 score of 0.5365. The RF model, with a 0.08 cutoff, had an AUROC of 0.7317, accuracy of 0.7730, precision of 0.5341, recall of 0.6507, and an F1 score of 0.5142. The LGBM model, at a 0.05 cutoff, showed an AUROC of 0.7490, accuracy of 0.8477, precision of 0.5405, recall of 0.6237, and an F1 score of 0.5440. At the end of this series, the ensemble model, which utilized a soft-voting technique with different weights of 0.02 for LGBM, 0.07 for XGBoost, 0.4 for CatBoost, 0.01 for RF, and 0.5 for MLP, delivered the best overall performance. It achieved an AUROC of 0.7944, accuracy of 0.9368, precision of 0.5821, recall of 0.5712, and an F1 score of 0.5762.

**Table 4:** Performance analysis of AI models for predicting prolonged ICU stay in postoperative critically ill patients

Model (cutoff)	AUROC	Accuracy	Precision	Recall	F1 score
XGBoost (0.07)	0.7658	0.8376	0.5366	0.6184	0.5365
CatBoost (0.07)	0.7731	0.8506	0.5553	0.6747	0.5648
LGBM (0.05)	0.7490	0.8477	0.5405	0.6237	0.5440
Random Forest (0.08)	0.7317	0.7730	0.5341	0.6507	0.5142
MLP (0.11)	0.7600	0.8879	0.5573	0.6282	0.5719
Ensemble model* (0.14)	0.7944	0.9368	0.5821	0.5712	0.5762

XGBoost, eXtreme Gradient Boosting; CatBoost, Categorical Boosting; LGBM, Light Gradient Boosting Machine; MLP, multilayer perceptron

^*^Using a soft-voting technique, the five models were assigned the following weights: 0.02 for LGBM, 0.07 for XGBoost, 0.4 for CatBoost, 0.01 for Random Forest, and 0.5 for MLP

Table 5; Fig. 3B present the external validation results using data from Center B. The CatBoost model showed an AUROC of 0.7294, accuracy of 0.8475, precision of 0.5359, recall of 0.6062, and an F1 score of 0.5380. The XGBoost model had an AUROC of 0.7214, accuracy of 0.8350, precision of 0.5263, recall of 0.5820, and an F1 score of 0.5221. The MLP model displayed an AUROC of 0.7038, accuracy of 0.9142, precision of 0.5579, recall of 0.5835, and an F1 score of 0.5672. The LGBM model had an AUROC of 0.6866, accuracy of 0.8550, precision of 0.5231, recall of 0.5614, and an F1 score of 0.5221. The RF model had an AUROC of 0.6687, accuracy of 0.9251, precision of 0.5285, recall of 0.5273, and an F1 score of 0.5279. At the conclusion of this validation, the ensemble model once again demonstrated superior performance, achieving an AUROC of 0.7376, accuracy of 0.9028, precision of 0.5564, recall of 0.5997, and an F1 score of 0.5689. The consistent performance of the ensemble model across the internal and external validations highlights its robustness and efficacy in predicting prolonged ICU stays.

**Table 5:** Cross-center validation of models for prolonged ICU stay prediction in postoperative critically ill patients

Model (cutoff)	AUROC	Accuracy	Precision	Recall	F1 score
XGBoost (0.08)	0.7214	0.8350	0.5263	0.5820	0.5221
CatBoost (0.07)	0.7294	0.8475	0.5359	0.6062	0.5380
LGBM (0.06)	0.6866	0.8550	0.5231	0.5614	0.5221
Random Forest (0.05)	0.6687	0.9251	0.5285	0.5273	0.5279
MLP (0.15)	0.7038	0.9142	0.5579	0.5835	0.5672
Ensemble model* (0.11)	0.7376	0.9028	0.5564	0.5997	0.5689

XGBoost, extreme Gradient Boosting; CatBoost, Categorical Boosting; LGBM, Light Gradient Boosting Machine; MLP, multilayer perceptron

^*^Using a soft-voting technique, the five models were assigned the following weights: 0.02 for LGBM, 0.07 for XGBoost, 0.4 for CatBoost, 0.01 for Random Forest, and 0.5 for MLP

### SHAP results of mortality and prolonged ICU stay prediction models

We analyzed feature impact using the SHAP method for 88 input variables. Red indicates a positive correlation with mortality or prolonged ICU stay, whereas blue indicates a negative correlation.

**Fig. 4:**
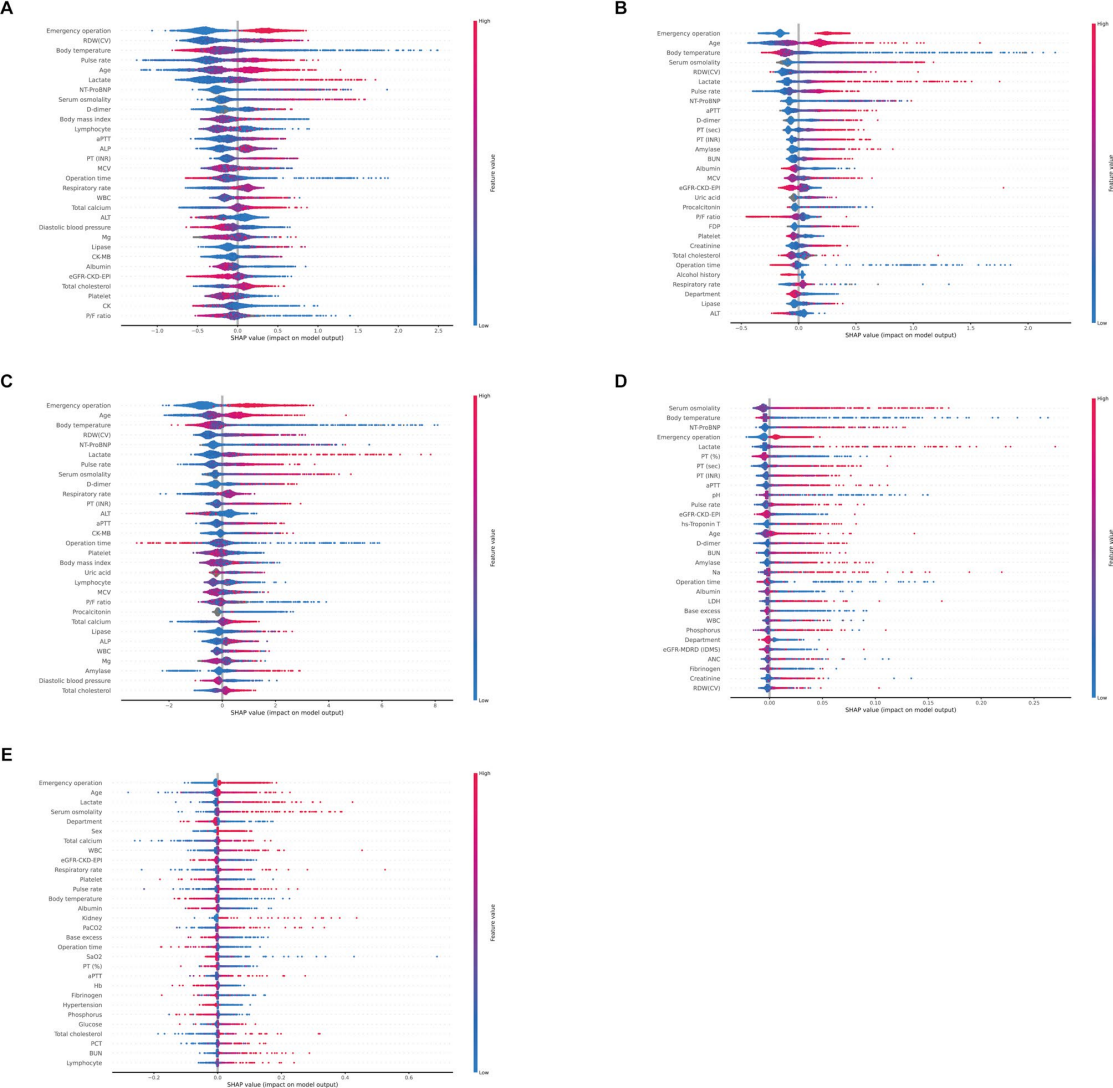
SHAP analysis of feature impact on mortality prediction across models. **A** eXtreme gradient boosting. **B** Category boosting. **C** Light gradient boosting machine. **D** Random forest. **E** Multilayer perceptron

Figure 4 shows the analysis of parameters affecting mortality prediction. In the XGBoost model, “Emergency surgery,” “Temperature,” and “Pulse rate” emerged as important features for predicting mortality. Laboratory tests such as “Red cell distribution width-coefficient of variation (RDW [CV]),” “Lactate,” “N-terminal pro-B-type natriuretic peptide (NT-proBNP),” and “Serum osmolality” were also significant contributors.

In the CatBoost model, “Emergency surgery,” “Age,” and “Temperature” were highlighted as key features. Important laboratory results included “Serum osmolality,” “RDW (CV),” “Lactate,” and “NT-proBNP,” aligning with the top parameters identified by XGBoost.

Similarly, the LGBM model identified “Emergency surgery,” “Age,” and “Body temperature” as prominent features. Laboratory markers such as “RDW (CV),” “NT-proBNP,” “Lactate,” and “Serum osmolality” also ranked highly, consistent with the findings from XGBoost and CatBoost.

In the SHAP analysis of the RF model, “Serum osmolality” was the most important feature, differing from the previous three models. However, “Temperature,” “NT-ProBNP,” “Emergency surgery,” and “Lactate” remained highly relevant, although their rankings varied.

In the MLP deep learning model, “Emergency Surgery,” “Age,” and “Department” were critical features. Important laboratory findings included “Lactate,” “Serum osmolality,” “Total calcium,” and “White blood cell count.” The SHAP results of the MLP model slightly differed from those of the machine-learning model.

In summary, common features influencing mortality prediction across the models included “Emergency surgery,” “Temperature,” “Serum osmolality,” “Lactate,” “RDW (CV),” and “NT-proBNP,” emphasizing their importance in postoperative critical care.

**Fig. 5:**
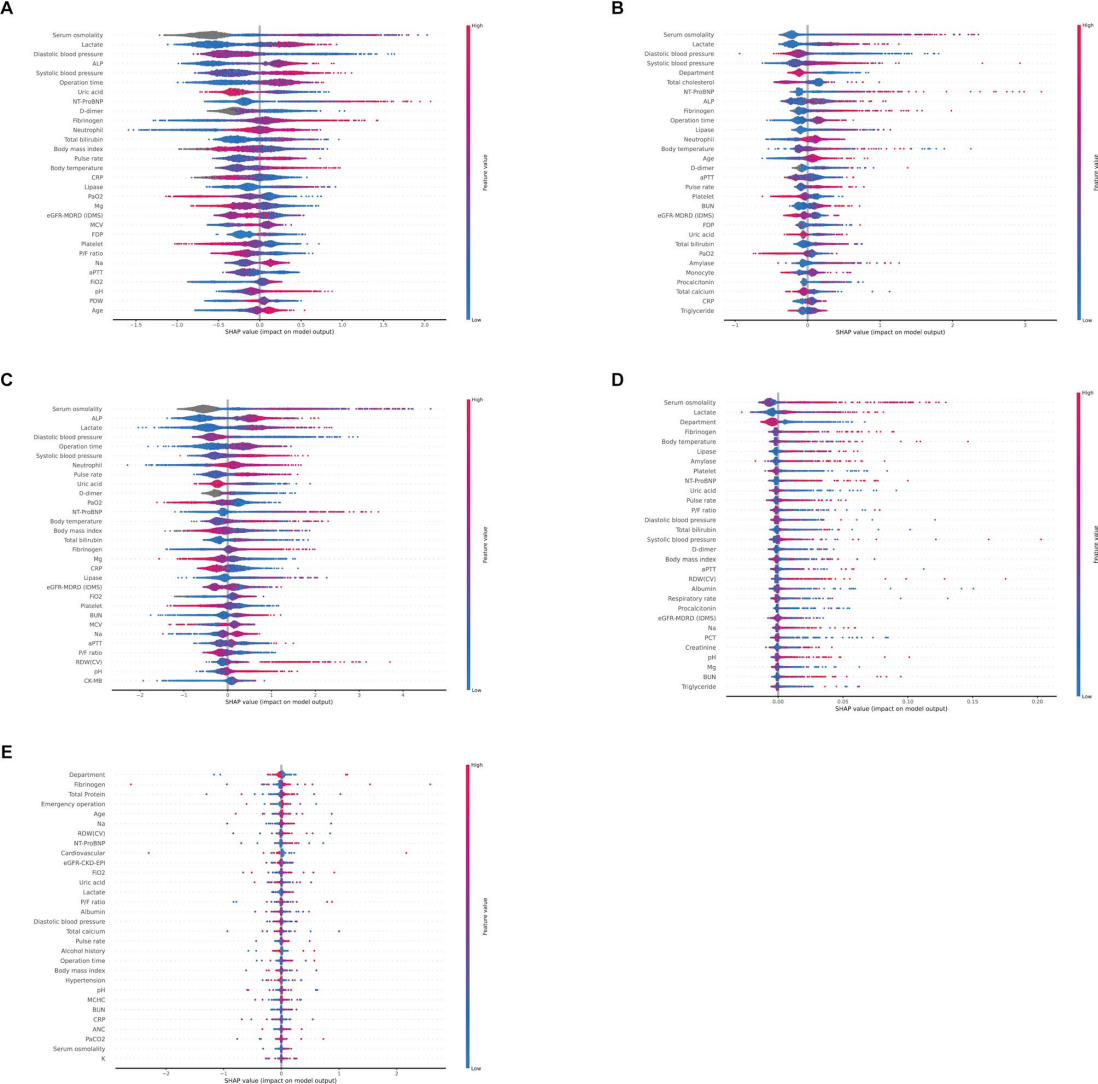
SHAP analysis of feature impact on prolonged ICU stay across models. **A** eXtreme gradient boosting. **B** Category boosting. **C** Light gradient boosting machine. **D** Random forest. **E** Multilayer perceptron

Figure 5 illustrates the SHAP analysis of features affecting prolonged ICU stay prediction. In the XGBoost model, “Serum osmolality,” “Lactate,” and “Diastolic blood pressure” emerged as prominent features, highlighting their predictive value for prolonged ICU stay. Similarly, in the CatBoost model, “Serum osmolality” ranked highest, with “Lactate” and “Diastolic blood pressure” also playing key roles.

The LGBM model confirmed the prominence of “Serum osmolality,” followed by “Alkaline phosphatase” and “Lactate.” These findings align with the results from the XGBoost and CatBoost models, reinforcing the importance of these laboratory tests in assessing long-term ICU risk.

The RF model similarly identified “Serum osmolality” and “Lactate” as influential factors along with “Department.” This further supports the trend observed in the other models, in which “Serum osmolality” and “Lactate” consistently ranked high.

Finally, in the MLP model, “Department” and “Fibrinogen” ranked highest, followed by “Total protein,” “Emergency surgery,” and “Age” in order of importance. Although “Serum osmolality” was less prominent in the MLP model, its consistent significance in other models highlighted its overall predictive value for ICU LOS.

Across all five models, “Serum osmolality,” “Lactate,” and “Diastolic blood pressure” repeatedly emerged as key features, confirming their robustness as predictors of prolonged ICU stay.

## Discussion

This study presents a novel and clinically valuable approach to predicting mortality and prolonged ICU stay in critically ill postoperative patients using advanced AI techniques. The significance of this model lies in its specificity for this unique patient population, which has distinct risk factors and pathophysiological profiles compared to the general ICU population. Existing mortality prediction models, such as APACHE, SAPS, and SOFA, provide valuable insights but are primarily designed for general ICU patients, potentially limiting their applicability in the postoperative setting.

We utilized a comprehensive multicenter dataset to ensure a broad and externally validated approach. This dataset enabled our model to capture a wide range of patient characteristics and outcomes, enhancing its utility across diverse clinical settings. In addition, using an ensemble approach that combines multiple machine-learning models showed superior predictive performance compared with individual models. This ensemble technique leverages the strengths of various algorithms, improving the accuracy and reliability of the predictions.

Accurately predicting mortality in critically ill patients is crucial, as it directly impacts patient care and resource allocation. Traditional severity scores such as the APACHE and SAPS typically achieve 70–80% accuracy in predicting mortality [8, 9]. However, these scores are not tailored for postoperative patients and may overlook unique factors that influence surgical outcomes. Postoperative critically ill patients face challenges such as surgical stress, interactions between preexisting comorbidities, and acute physiologic changes resulting from surgery. These complexities necessitate specialized models such as those developed in this study to predict mortality more accurately in this specific patient group. High-performance mortality prediction models can help clinicians identify high-risk patients early, enabling timely interventions and personalized treatment plans. Accurate prediction of mortality is also essential for optimizing healthcare resource allocation. Effective management of ICU beds, ventilators, and medical staff ensures that patients receive the appropriate level of care. Conversely, inaccurate forecasting can lead to under- or over-utilization of resources, posing significant financial and operational implications for healthcare facilities.

Predicting prolonged ICU stays has been under-researched but is critically important. Although mortality forecasting focuses on the ultimate outcome, ICU LOS prediction addresses the duration of intensive care required by a patient. Longer ICU stays are associated with more complex clinical needs, including an increased risk of complications, longer hospital stays, and higher healthcare costs. Identifying patients at risk for extended ICU stays allows for better resource allocation and management. Healthcare facilities can plan long-term care, deploy specialized staff, and adjust bed availability accordingly. This proactive approach improves patient care while optimizing the utilization of limited ICU resources. Globally, critical care resources are scarce, and during the pandemic, they have been stretched to an all-time high [20–22]. In addition, predicting prolonged ICU stays enables clinicians to tailor treatment plans and interventions to meet patients’ specific needs. This personalized approach can lead to better outcomes, fewer complications, and shorter hospital stays, ultimately benefiting patients and the healthcare system.

This study highlights the effectiveness of ensemble techniques in predictive modeling. Ensemble models combine predictions from multiple individual models to increase overall accuracy and reduce overfitting [23–25]. In our study, the ensemble model outperformed individual models in predicting mortality and ICU LOS. Ensemble models are particularly useful in healthcare applications because they capture a wide range of data patterns and complexities. Each individual model has its strengths and weaknesses, and ensemble techniques capitalize on these differences to provide more robust predictions. By combining XGBoost, LGBM, CatBoost, RF, and MLP into an ensemble model, we leveraged the strengths of each algorithm. We developed an ensemble model using a soft-voting technique, which combines predictions from multiple individual models, leveraging their diverse strengths and mitigating their weaknesses. This ensemble approach typically results in improved prediction accuracy, robustness, and generalization performance compared to individual models. Soft-voting assigns weights to the predictions of each model, enabling a more flexible and balanced integration of their outputs. This not only enhances the predictive power but also helps reduce overfitting. Overall, soft-voting ensemble models provide a more reliable and stable framework for making complex predictions, making them valuable for various machine-learning applications. The ensemble approach also improves the generalizability of the model, as demonstrated by its consistent performance during external validation. This means the model is not limited to the specific patient population or clinical setting in which it was trained and can be effectively applied across diverse healthcare environments. Our ensemble model represents a significant advancement in predictive modeling for postoperative critically ill patients. The model addresses the pressing need for effective critical care and healthcare resource management by providing clinicians with a powerful tool for predicting mortality and ICU LOS. The combination of advanced AI techniques, extensive datasets, and ensemble modeling offers immense potential for improving patient outcomes and optimizing ICU care.

The SHAP results from the mortality prediction model provide important insights into the parameters significantly impacting patient outcomes. Among these, emergency surgery consistently emerged as a crucial predictor of mortality owing to its urgent and often complex nature. Emergency surgery is associated with significantly higher morbidity and mortality rates than elective or routine surgery. Several factors influence outcomes after emergency surgery, including the patient’s preoperative clinical condition, availability of medical resources, and the efficiency of administrative and organizational processes [26–28]. A study of emergency general surgery patients in the UK National Health Service revealed that these patients, who accounted for more than one-third of hospital admissions, had a mortality rate approximately eight times higher than that of those undergoing elective surgery. This stark difference highlights the high risks associated with emergency surgery [29]. Another important consideration is the 30-day postoperative outcomes. For emergency surgery, primary outcomes such as 30-day mortality and secondary outcomes such as complications, reoperation, and readmission rates are important metrics. These rates differ significantly between urgent and emergency cases versus elective surgery [30]. In addition, certain types of emergency surgery are particularly high risk; for example, emergency laparoscopic surgery is considered high risk, with a mortality rate exceeding 20% [31]. The complexity and urgency of these surgeries contribute to increased mortality risk. “Temperature” and “Lactate” levels were also highlighted as important markers, reflecting their roles in indicating systemic inflammation and metabolic stress, respectively. Elevated lactate levels are a significant indicator of outcomes in critically ill patients, especially in postoperative cases. Serum lactate levels indicate an imbalance between oxygen supply and demand resulting from circulatory impairment. In critically ill patients, hyperlactatemia often results from tissue hypoxia caused by anaerobic glycolysis, making it a strong predictor of mortality not only in this population but also specifically in the postoperative context [32]. Similarly, “NT-proBNP” and “RDW (CV)” have been identified as important factors. In a study of critically ill patients, the median NT-proBNP levels were significantly higher in those who died than in survivors (11,859 ng/L versus 2,534 ng/L), indicating that elevated NT-proBNP levels can predict mortality [33]. RDW (CV), commonly measured as the coefficient of variation of red blood cell size, is often undervalued in critical care. However, evidence suggests that RDW is associated with mortality in mixed cohorts of critically ill patients. One study revealed that an elevated RDW at ICU admission was associated with a higher 90-day mortality rate in surgical patients, suggesting its potential as a marker for early risk stratification [34]. Interestingly, “Operative time,” traditionally considered an important factor in postoperative outcomes, was found to be less influential than the urgency of surgery. Table 1 shows that emergency surgeries generally had shorter operative times despite their higher risks. This suggests a complex relationship among operative time, urgency of surgery, and patient outcomes. Considering the context and nature of the surgery, rather than focusing solely on operative time, is crucial when assessing patient outcomes. These findings challenge conventional wisdom and call for further exploration of how surgical factors contribute to critical care trajectories.

In the assessment of prolonged ICU stays, certain physiological parameters consistently feature across various predictive models. However, there is limited research on the parameters influencing prolonged ICU stays in the critical care domain. Nevertheless, we found that these parameters provide valuable insights. Serum osmolality is one such parameter that has repeatedly been identified as a key feature. A study on critically ill patients in the ICU revealed that higher serum osmolality upon admission was associated with an increased ICU mortality rate. In our study, serum osmolality positively correlated with ICU mortality rate. It was also highly associated with prolonged ICU stays, with SHAP results showing a U-shaped relationship, where very high and low values were associated with prolonged ICU stays. These results suggest that abnormal serum osmolality, whether high or low, indicates a severe or complex disease course that may lead to prolonged ICU stays [35].

An unusual finding in our study was the high importance of “Diastolic blood pressure” in the XGBoost and CatBoost models. Although limited research exists on the association between diastolic blood pressure and postoperative critical care, some studies have shown that intraoperative diastolic hypotension (defined as a diastolic blood pressure < 60 mm Hg for >10 min) in patients undergoing gastric cancer surgery is associated with significantly longer postoperative hospital stays and higher incidences of postoperative complications [36]. This suggests that intraoperative diastolic blood pressure may influence postoperative recovery and ICU LOS.

The relationship between lactate levels and prolonged ICU stays in postoperative critically ill patients is an important topic in critical care. Elevated lactate levels can be a cause and a consequence of critical illness and are often used as biomarkers for patient prognosis. In critically ill patients, particularly those who have undergone major surgeries, elevated lactate levels often indicate tissue hypoperfusion. This condition can result from various factors such as hemorrhage, sepsis, cardiac failure, or severe dehydration. Lactate is produced when cells switch to anaerobic metabolism as a result of insufficient oxygen supply [37, 38]. Elevated lactate levels can serve as predictive markers for prolonged ICU stays. Studies have shown that postoperative patients with high lactate levels often require extended intensive care, reflecting the severity and complexity of their conditions [39].

In MLP, a deep learning model, fibrinogen and total protein were identified as important parameters for predicting prolonged ICU stays. Fibrinogen, a key protein in the coagulation cascade, plays an important role in hemostasis and inflammatory processes [40]. Elevated fibrinogen levels after surgery may reflect a prolonged inflammatory response or an increased risk of thrombotic complications, both of which can extend ICU stay. Conversely, low fibrinogen levels may indicate a risk of bleeding complications, also contributing to prolonged ICU care [41, 42]. Although fibrinogen has been considered a non-specific biomarker, the findings of our study reaffirm its clinical relevance. The relationship between total protein levels and prolonged ICU stays in postoperative critically ill patients is of clinical interest, particularly in the context of nutritional status and its impact on recovery. However, total protein levels are often underestimated in clinical practice. Adequate protein levels are essential for wound healing and immune function. Postoperative patients with low protein levels may experience slower recovery, increased infection risk, and impaired wound healing, leading to prolonged ICU stays. Low total protein levels can result in complications such as edema, delayed wound healing, and increased susceptibility to infection, which contribute to longer ICU stays and more complex clinical outcomes [43, 44]. The results of our study highlight the importance of nutritional status in the management of critically ill patients after surgery using AI-based predictive models.

An important consideration is how this predictive tool can be integrated into clinical workflows. The variables required for prediction—laboratory results, vital signs, and operative information—are routinely collected at ICU admission and stored in electronic health records (EHRs). Thus, the model could be implemented within EHR systems to automatically extract these data and generate individualized risk scores at the bedside. Predictions could be displayed on clinician-facing dashboards within existing decision-support platforms, enabling rapid identification of high-risk patients. Importantly, because the model produces outputs within seconds once data are available, it has strong potential for real-time application. Such integration would support early triage, timely interventions, optimized ICU resource allocation, and improved interdisciplinary communication. Future prospective studies should evaluate the usability and impact of embedding this tool into real-world clinical workflows.

Although this study offers significant insights into the application of AI in critical care, it has some limitations. The choice of > 30 days as the threshold for prolonged ICU stay also warrants comment. Although many studies adopt shorter cutoffs such as 7 or 14 days, the 30-day threshold has strong clinical justification. It aligns with the established use of 30-day mortality as a benchmark outcome in surgical and ICU research and reflects real-world practice in Korea, where ICU stays beyond 30 days are formally classified and managed as long-term admissions. This definition highlights a subgroup of patients with extreme morbidity, poor outcomes, and disproportionate resource utilization. Nonetheless, alternative thresholds may also be informative, and future studies should examine model adaptability across varying definitions. Its retrospective design introduces the potential for inherent bias, emphasizing the need for prospective validation. Data collection from only two medical centers may limit the generalizability of the findings, as it may not fully reflect the diversity of clinical settings. Despite rigorous external validation, the study was conducted within a similar regional healthcare setting. Future research should include broader multicenter validation across different countries and healthcare systems to ensure the robustness and adaptability of the model in varied clinical environments. The dynamic nature of postoperative critical care also poses challenges for prediction accuracy, as rapid changes in patient status may not be fully captured by the model. In addition, handling missing data, particularly when using models that require data imputation, introduces complexity that can affect prediction reliability. Another limitation is the absence of calibration analyses such as calibration curves or Brier scores. Although discrimination was robust across validation cohorts, future work should include calibration assessment to evaluate the agreement between predicted probabilities and observed outcomes.

## Conclusions

This study represents a significant advancement in the application of AI in critical care, particularly for postoperative critically ill patients. By developing an ensemble model that integrates multiple machine-learning techniques, this study provides a tool capable of predicting mortality and prolonged ICU stays more accurately and robustly than existing models can. The model demonstrated strong performance in internal development and external validation, suggesting its potential utility in clinical practice. Additionally, the use of the SHAP method for functional impact analysis provides valuable insights into the factors influencing patient outcomes, thereby guiding clinicians to make more informed decisions. Our findings have important implications for patient care and optimizing ICU resource allocation and management—an urgent need in today’s healthcare environment.

## Supplementary Information

Below is the link to the electronic supplementary material.

**Additional file 1**: Comprehensive laboratory profile analysis of postoperative critically ill patients from two medical centers

## Data Availability

The datasets used and/or analyzed in the current study are available from the corresponding author upon reasonable request.

## Abbreviations

AI: Artificial intelligence
APACHE: Acute physiology and chronic health evaluation
AUROC: Area under the receiver operating characteristic curve
BMI: Body mass index
CatBoost: Categorical boosting
EHR: Electronic health record
ICU: Intensive care unit
LGBM: Light gradient boosting machine
LOS: Length of stay
MLP: Multilayer perceptron
NT-proBNP: N-terminal pro-B-type natriuretic peptide
RDW (CV): Red cell distribution width-coefficient of variation
RF: Random forest
SAPS: Simplified acute physiology score
SOFA: Sequential organ failure assessment
SHAP: SHapley Additive exPlanations
XGBoost: eXtreme gradient boosting
