# Correction: Development and external validation of an artificial intelligence model for predicting mortality and prolonged ICU stay in postoperative critically ill patients: a retrospective study

**DOI:** 10.1186/s13017-025-00663-x

**Published:** 2025-11-26

**Authors:** Dong Jin Park, Seung Min Baik, Kyung Sook Hong, Heejung Yi, Jae Gil Lee, Jae-Myeong Lee

**Affiliations:** 1 Department of Laboratory Medicine, Eunpyeong St. Mary’s Hospital, College of Medicine, The Catholic University of Korea, Seoul, Korea; 2 Division of Critical Care Medicine, Department of Surgery, Ewha Womans University Mokdong Hospital, Ewha Womans University College of Medicine, Seoul, Korea; 3 Department of Surgery, Korea University College of Medicine, Seoul, Korea; 4 Division of Critical Care Medicine, Department of Surgery, Ewha Womans University Seoul Hospital, Ewha Womans University College of Medicine, Seoul, Korea; 5 Division of Acute Care Surgery, Department of Surgery, Korea University Anam Hospital, Korea University College of Medicine, Goryeodae-ro 73, Seongbuk-gu, Seoul, 02841 Republic of Korea

**Correction to: World Journal of Emergency Surgery (2025) 20:79**

10.1186/s13017-025-00650-2

In this article [1], Figs. 2, 3, 4 and 5 appeared incorrectly with black background and have now been corrected in the original publication. The figure should have appeared as shown below.

**Fig. 2:**
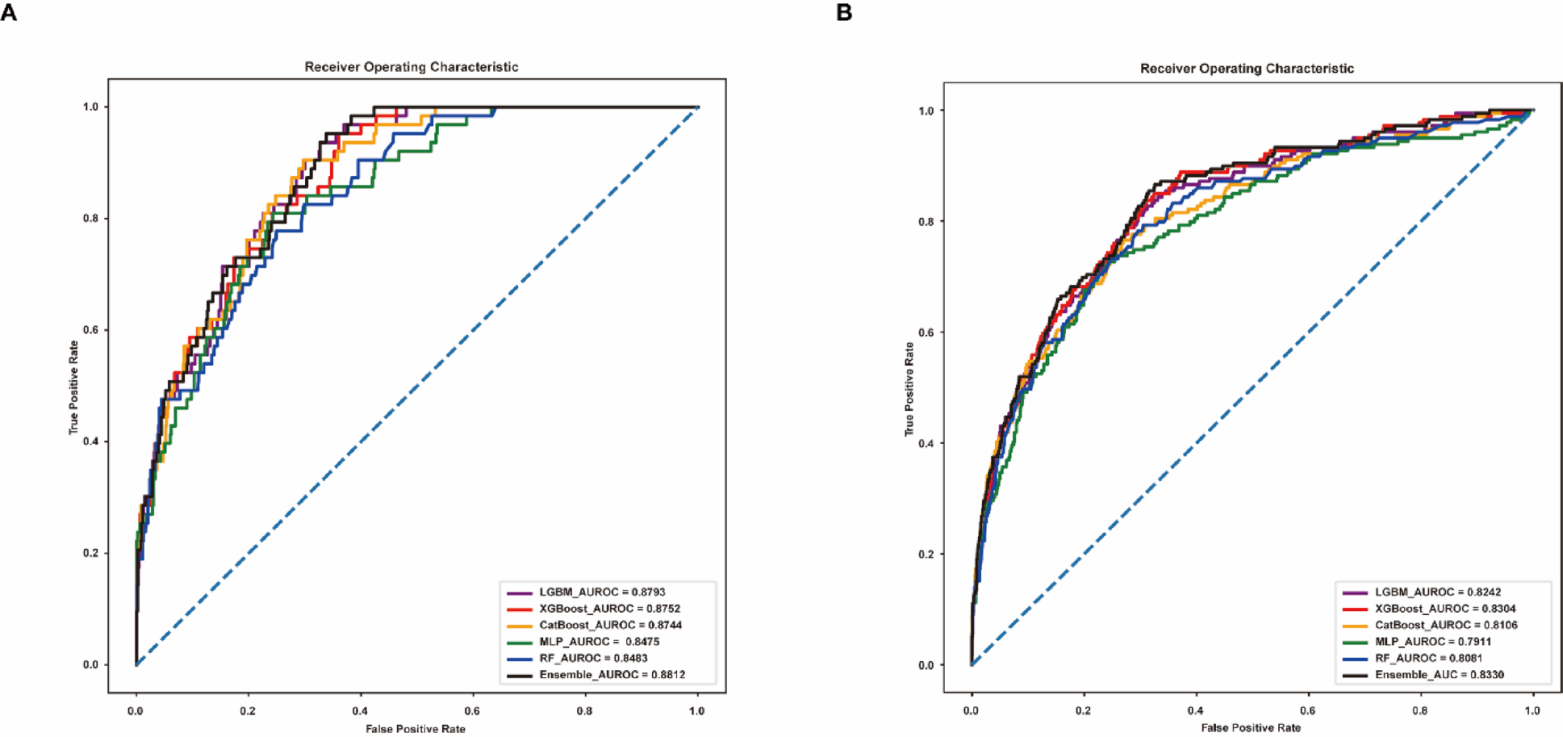
Area Under the Receiver Operating Characteristic Curve for Mortality Predictions of the Models. **A** AUROC of the initial model. **B** AUROC of the externally validated model. AUROC, area under the receiver operating characteristic curve

**Fig. 3:**
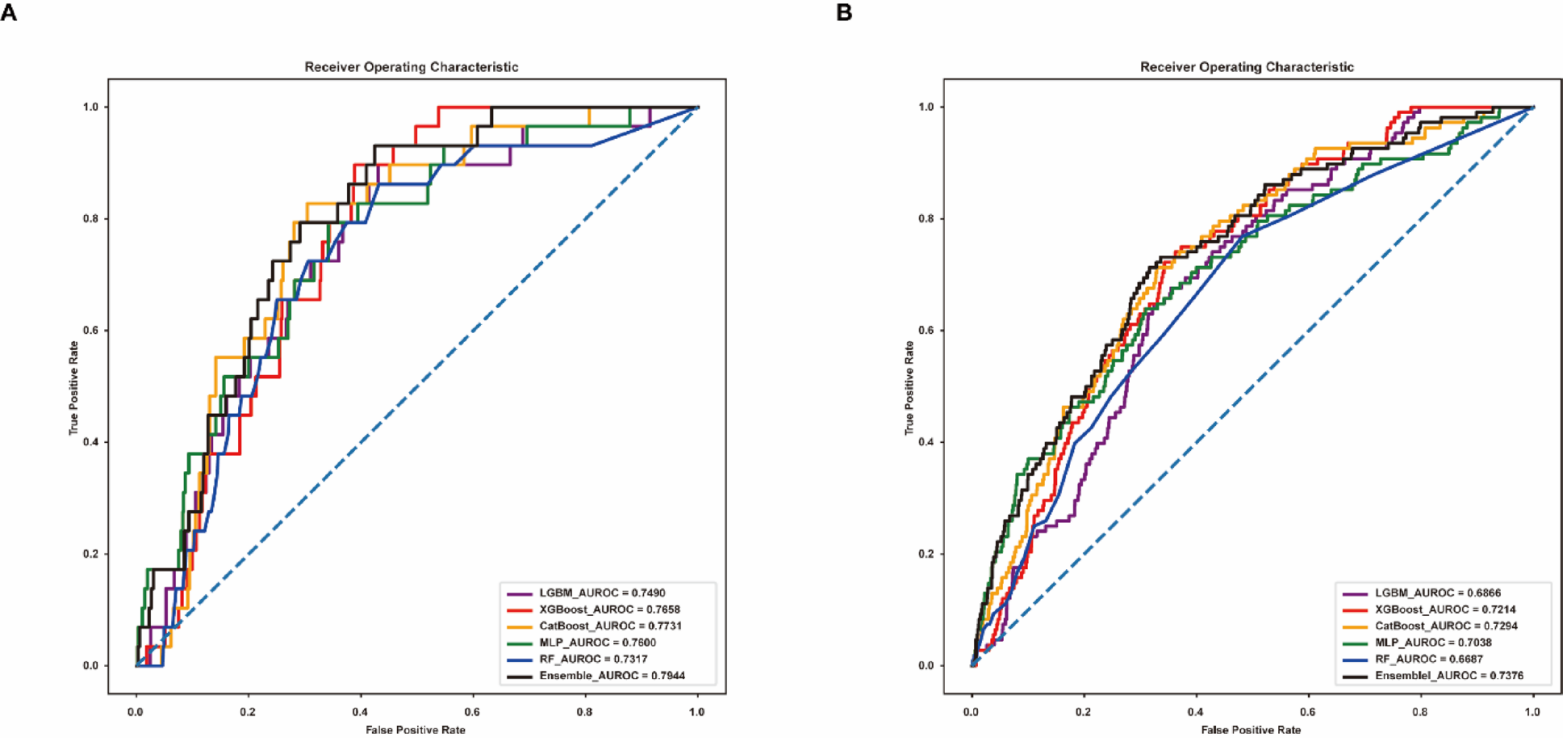
Area Under the Receiver Operating Characteristic Curve for Prolonged ICU Stay Predictions of the Models. **A** AUROC of the initial model. **B** AUROC of the externally validated model. AUROC, area under the receiver operating characteristic curve

**Fig. 4:**
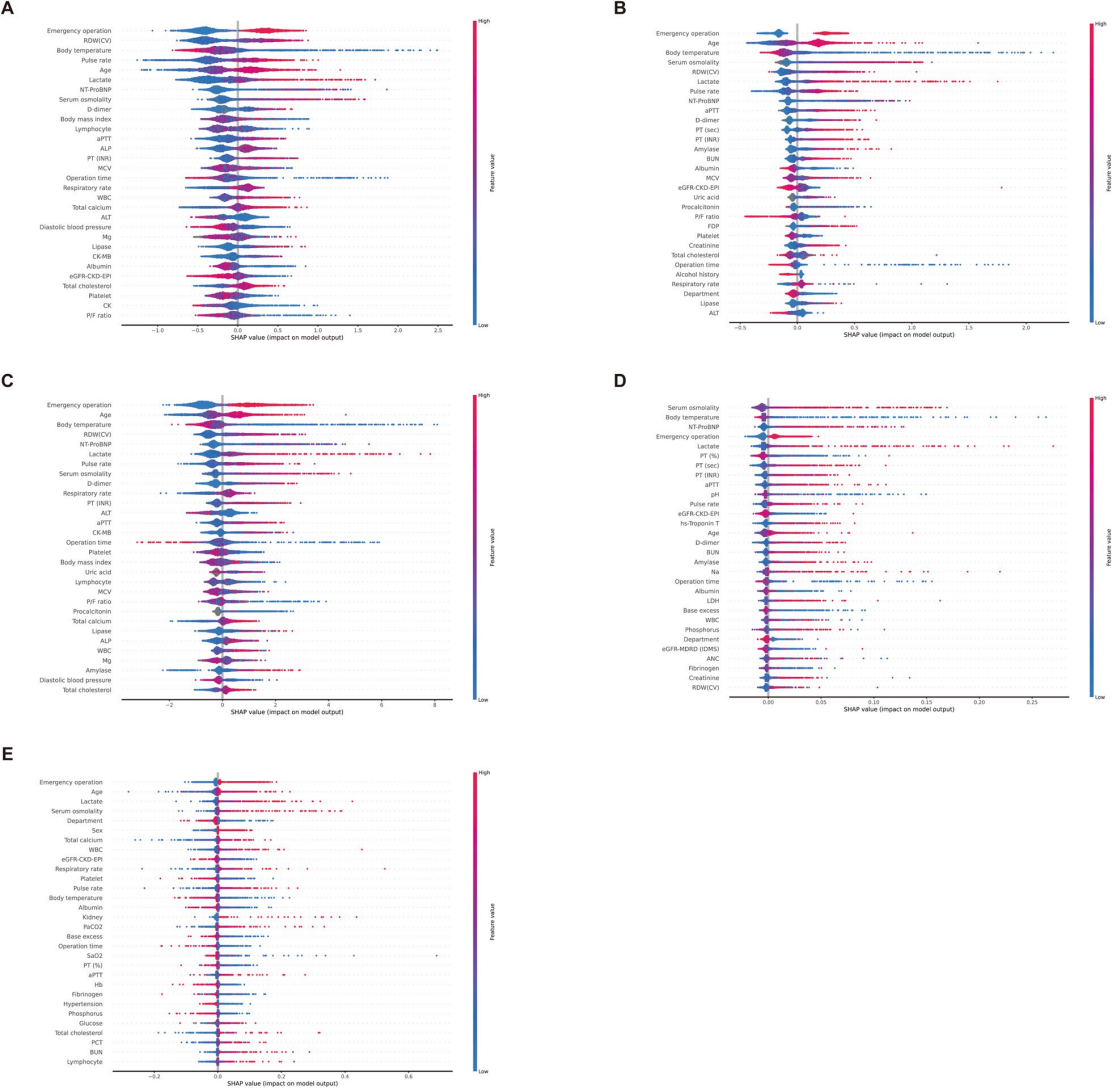
SHAP Analysis of Feature Impact on Mortality Prediction Across Models. **A** eXtreme Gradient Boosting. **B** Category Boosting. **C** Light Gradient Boosting Machine. **D** Random Forest. **E** Multilayer perceptron

**Fig. 5:**
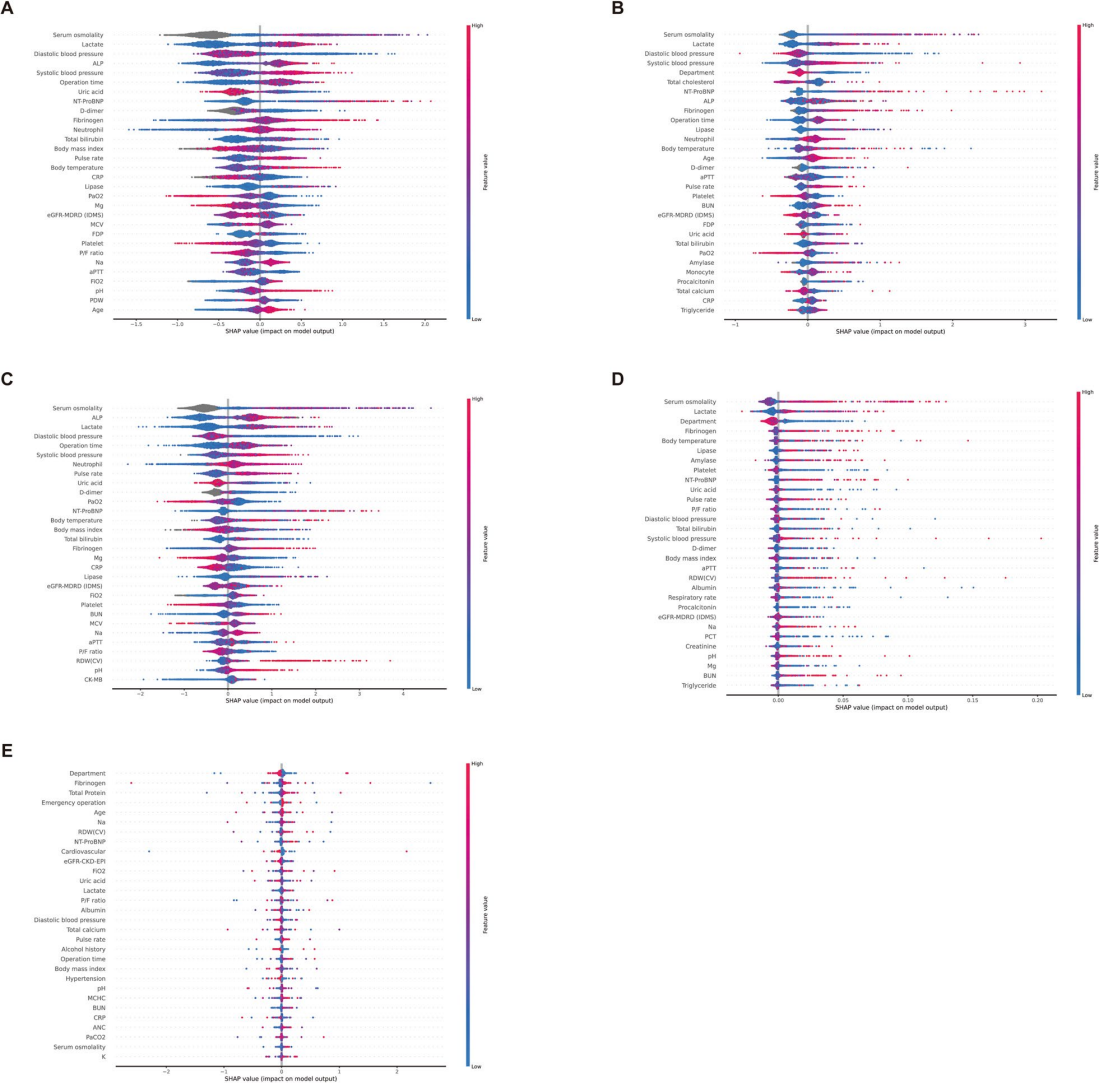
SHAP Analysis of Feature Impact on Prolonged ICU Stay Across Models. **A** eXtreme Gradient Boosting. **B** Category Boosting. **C** Light Gradient Boosting Machine. **D** Random Forest. **E** Multilayer perceptron
