# Supplementary material for: Development and external validation of an artificial intelligence model for predicting mortality and prolonged ICU stay in postoperative critically ill patients: a retrospective study
Source: World J Emerg Surg. 2025 Oct 15;20:79. doi: 10.1186/s13017-025-00650-2 (PMC12523136; doi:10.1186/s13017-025-00650-2)
Supplement: Supplementary file 1 — Additional file 1: Comprehensive laboratory profile analysis of postoperative critically ill patients from two medical centers [file 13017_2025_650_MOESM1_ESM.docx]

**Supplementary Table 1.** Comprehensive Laboratory Profile Analysis of Postoperative Critically Ill Patients from Two Medical Centers

|  | **Center A** |  |  | **Center B** |  |  |
| --- | --- | --- | --- | --- | --- | --- |
| **Variables** | **Survival group** | **Deceased group** | ***P*** | **Survival group** | **Deceased group** | ***P*** |
| White blood cell count (×10^3^/uL) | 11.80 ± 5.74 | 12.79 ± 9.23 | 0.065 | 12.24 ± 5.97 | 12.88 ± 9.09 | 0.362 |
| Red blood cell count (×10^6^/uL) | 3.58 ± 0.71 | 3.33 ±  0.80 | <0.001 | 3.61 ± 0.67 | 3.33 ± 0.82 | <0.001 |
| Hemoglobin (g/dL) | 10.9 ± 2.2 | 10.2 ±  2.4 | <0.001 | 11.1 ± 2.1 | 10.2 ± 2.6 | <0.001 |
| Hematocrit (%) | 31.7 ± 6.1 | 29.7 ±  6.9 | <0.001 | 32.8 ± 6.1 | 30.3 ± 7.5 | <0.001 |
| Mean corpuscular volume (fL) | 88.8 ± 5.6 | 89.7 ±  5.9 | 0.009 | 91.1 ± 5.9 | 91.5 ± 6.6 | 0.391 |
| Mean corpuscular hemoglobin (pg) | 30.5 ± 2.3 | 30.7 ± 2.2 | 0.191 | 30.8 ± 2.4 | 30.7 ± 2.6 | 0.628 |
| Mean corpuscular hemoglobin concentration (%) | 34.4 ± 1.3 | 34.2 ± 1.3 | 0.073 | 33.8 ± 1.0 | 33.6 ± 1.2 | 0.001 |
| Red cell distribution width | 13.9 ± 2.2 | 14.5 ± 2.2 | <0.001 | 14.4 ± 2.3 | 15.2 ± 3.0 | 0.002 |
| Platelet (×10^3^/uL) | 195.6 ± 90.0 | 163.7 ± 95.9 | <0.001 | 205.6 ± 94.3 | 175.8 ± 101.9 | <0.001 |
| Plateletcrit (%) | 0.20 ± 0.09 | 0.17 ± 0.09 | <0.001 | 0.17 ± 0.07 | 0.15 ± 0.09 | 0.030 |
| Mean Platelet Volume (fL) | 10.3 ± 1.0 | 10.4 ± 1.1 | 0.001 | 10.0 ± 0.9 | 10.2 ± 1.1 | 0.005 |
| Platelet volume distribution width (fL) | 11.4 ± 2.2 | 11.7 ± 2.5 | 0.017 | 16.7 ± 1.5 | 16.4 ± 2.5 | 0.212 |
| Absolute neutrophil count (×10^3^/uL) | 10.0 ± 5.4 | 10.5 ± 7.0 | 0.175 | 10.4 ± 5.7 | 10.7 ± 6.4 | 0.521 |
| Neutrophil (%) | 82.5 ±9.1 | 81.5 ± 12.4 | 0.161 | 82.2 ± 9.8 | 82.9 ± 11.0 | 0.364 |
| Lymphocyte (%) | 10.4 ± 6.9 | 11.2 ± 9.4 | 0.139 | 10.5 ± 7.7 | 10.3 ± 8.3 | 0.757 |
| Monocyte (%) | 6.2 ± 3.2 | 6.5 ± 6.1 | 0.337 | 6.3 ± 3.2 | 5.9 ± 4.6 | 0.190 |
| Eosinophil (%) | 0.7 ± 2.1 | 0.5 ± 1.1 | 0.198 | 0.8 ± 1.8 | 0.6 ± 1.2 | 0.220 |
| Basophil (%) | 0.3 ± 0.2 | 0.3 ± 0.3 | 0.276 | 0.4 ± 0.3 | 0.3 ± 0.4 | 0.563 |
| Activated Partial Thromboplastin Time (sec) | 29.8 ± 12.5 | 40.1 ± 29.7 | <0.001 | 30.9 ± 13.8 | 43.0 ± 32.2 | <0.001 |
| Prothrombin Time (sec) | 14.0 ± 5.0 | 16.5 ± 5.2 | <0.001 | 14.1 ± 3.1 | 18.6 ± 15.1 | <0.001 |
| Prothrombin Time (%) | 78.8 ± 19.2 | 63.8 ± 22.3 | <0.001 | 76.0 ± 17.8 | 61.7 ± 23.6 | <0.001 |
| Prothrombin Time (INR) | 1.20 ± 0.33 | 1.43 ± 0.47 | <0.001 | 1.21 ± 0.27 | 1.51 ± 0.69 | <0.001 |
| Total calcium (mg/dL) | 7.9 ± 0.7 | 7.8 ± 0.9 | 0.088 | 8.2 ± 0.7 | 7.8 ± 1.0 | <0.001 |
| Phosphorus (mg/dL) | 3.3 ± 1.1 | 4.0 ± 2.2 | <0.001 | 3.4 ± 1.0 | 3.9 ± 2.1 | 0.003 |
| Glucose (mg/dL) | 156 ± 56 | 173 ± 74 | <0.001 | 162 ± 52 | 186 ± 70 | <0.001 |
| Blood urea nitrogen (mg/dL) | 19.6 ± 15.1 | 27.4 ± 20.8 | <0.001 | 20.5 ±16.4 | 27.0 ±21.3 | <0.001 |
| Creatinine (mg/dL) | 1.12 ± 1.36 | 1.66 ± 1.67 | <0.001 | 1.09 ±1.35 | 1.36 ±1.27 | 0.012 |
| eGFR-MDRD -IDMS (mL/min/1.73 m2) | 88.3 ± 41.5 | 68.3 ± 44.9 | <0.001 | 97.9 ± 50.5 | 80.5 ±60.0 | <0.001 |
| eGFR-CKD-EPI (mL/min/1.73 m2) | 79.6 ± 29.2 | 62.7 ± 33.3 | <0.001 | 82.5 ± 30.9 | 68.5 ±35.6 | <0.001 |
| Triglyceride (mg/dL) | 103.3 ± 62.8 | 109.8 ± 74.5 | 0.189 | 96.72 ± 55.23 | 120.6 ±119.1 | 0.053 |
| Total cholesterol (mg/dL) | 109.6 ± 37.8 | 97.0 ± 42.2 | <0.001 | 114.70 ± 42.5 | 95.0 ± 49.5 | <0.001 |
| Total protein (g/dL) | 5.5 ± 0.9 | 5.1 ± 1.1 | <0.001 | 5.6 ± 0.9 | 5.2 ± 1.3 | <0.001 |
| Albumin (g/dL) | 3.2 ± 0.6 | 2.9 ± 0.7 | <0.001 | 3.2 ±0.6 | 2.9 ± 0.8 | <0.001 |
| Aspartate aminotransferase (IU/L) | 78.8 ± 330.7 | 192.9 ± 823.4 | 0.016 | 72.1 ±148.3 | 213.7 ± 804.3 | 0.024 |
| Alanine aminotransferase (IU/L) | 51.9 ± 154.0 | 144.4 ± 854.3 | 0.059 | 49.7 ± 114.7 | 118.2 ± 413.6 | 0.034 |
| Alkaline phosphatase (IU/L) | 133.6 ± 142.2 | 154.0 ± 169.2 | 0.084 | 937 ±106.0 | 121.1 ±154.3 | 0.081 |
| Total bilirubin (mg/dL) | 1.27 ± 2.78 | 1.53 ± 3.89 | 0.261 | 1.29 ±1.85 | 2.31 ± 5.53 | 0.061 |
| Amylase (IU/L) | 114.8 ± 674.4 | 187.9 ± 313.7 | 0.112 | 75.6 ± 138.8 | 156.9 ± 290.9 | 0.018 |
| Lipase (U/L) | 96.2 ± 1254.4 | 70.4 ± 133.9 | 0.760 | 49.6 ± 168.7 | 96.8 ± 395.8 | 0.309 |
| Sodium (mmol/L) | 138.4 ± 4.1 | 140.1 ± 6.6 | <0.001 | 136.6 ± 4.1 | 138.2 ± 5.7 | <0.001 |
| Potassium (mmol/L) | 4.1 ± 0.6 | 4.2 ± 0.8 | 0.061 | 4.0 ± 0.5 | 4.1 ± 0.8 | 0.226 |
| Chloride (mmol/L) | 104.3 ± 4.7 | 104.5 ± 6.8 | 0.520 | 105.6 ± 4.8 | 105.3 ± 5.6 | 0.602 |
| Magnesium (mg/dL) | 2.14 ± 0.34 | 2.25 ± 0.51 | <0.001 | 2.09 ± 0.35 | 2.24 ± 0.45 | <0.001 |
| Total carbon dioxide (mmol/L) | 20.2 ± 3.4 | 18.4 ± 4.4 | <0.001 | 21.5 ±3.6 | 19.5 ± 4.8 | <0.001 |
| C-reactive protein (mg/dL) | 8.9 ± 8.9 | 10.5 ± 10.3 | 0.011 | 6.8 ± 8.2 | 9.4 ± 10.1 | 0.005 |
| Creatine kinase (IU/L) | 461.0 ± 1435.2 | 522.2 ± 892.6 | 0.543 | 317.4 ± 1083.1 | 639.2 ± 1615.8 | 0.060 |
| Creatine kinase-MB (ng/mL) | 8.17 ± 24.96 | 13.61 ± 28.93 | 0.009 | 7.29 ±27.65 | 17.77 ± 62.93 | 0.109 |
| High sensitivity-Troponin T (ng/mL) | 0.170 ± 0.836 | 0.358± 1.081 | 0.015 | 0.163 ±1.144 | 0.543 ± 2.549 | 0.149 |
| Procalcitonin (ng/mL) | 9.47 ± 18.62 | 13.01 ± 20.77 | 0.122 | 5.47 ±15.17 | 12.70 ± 23.40 | 0.003 |
| Lactate dehydrogenase (IU/L) | 340.1 ± 561.8 | 914.5 ± 2196.0 | 0.001 | 274.4 ±183.2 | 442.7 ± 464.2 | 0.002 |
| N-terminal pro-B-type natriuretic peptide (ng/L) | 3713.8 ± 8376.1 | 8563.7 ± 13448.4 | <0.001 | 3320.3 ± 9754.4 | 4908.8 ± 10901.3 | 0.149 |
| Uric acid (mg/dL) | 4.5 ± 2.1 | 5.0 ± 2.2 | 0.008 | 4.6 ± 2.3 | 6.3 ± 3.1 | <0.001 |
| Serum osmolarity (mOsm/kg) | 292.4 ± 14.4 | 304.2 ± 17.6 | <0.001 | 289.8 ± 11.1 | 299.4 ± 17.8 | <0.001 |
| Ferritin (ng/mL) | 1802.7 ± 6334.7 | 13289.7 ± 32643.4 | 0.323 | 699.0 ± 1058.4 | 465.0 ±577.7 | 0.596 |
| Fibrinogen (mg/dL) | 363.0 ± 162.0 | 322.7 ± 184.7 | 0.004 | 340.6 ± 152.3 | 272.2 ±161.5 | 0.001 |
| Fibrinogen degradation production (ug/mL) | 19.0 ± 21.4 | 28.2 ± 26.2 | <0.001 | 17.5 ± 21.9 | 33.6 ± 30.3 | <0.001 |
| D-dimer (mg/L) | 6.84 ± 8.57 | 10.79 ± 11.09 | <0.001 | 6.34 ± 7.40 | 10.91 ± 10.81 | 0.001 |
| Arterial blood gas analysis |  |  |  |  |  |  |
| pH | 7.396 ± 0.678 | 7.344 ± 0.123 | <0.001 | 7.388 ± 0.068 | 7.361 ± 0.117 | 0.004 |
| PaCO_2_ (mmHg) | 36.7 ± 5.9 | 37.6 ± 10.3 | 0.114 | 37.5 ±6.5 | 36.5 ± 9.1 | 0.187 |
| PaO_2_ (mmHg) | 157.7 ± 82.0 | 169.0 ± 106.3 | 0.078 | 149.0 ± 70.1 | 160.6 ± 92.0 | 0.123 |
| Bicarbonate (mmol/L) | 22.1 ± 3.5 | 20.0 ± 4.5 | <0.001 | 22.1 ± 3.3 | 20.2 ± 4.6 | <0.001 |
| Base excess (mmol/L) | -2.71 ± 4.14 | -5.62 ± 5.86 | <0.001 | -2.92 ± 3.91 | -5.26 ± 5.85 | <0.001 |
| CO_2_ content (mmol/L) | 23.2 ± 3.7 | 21.2 ± 4.6 | <0.001 | 23.2 ± 3.4 | 21.3 ± 4.8 | <0.001 |
| O_2_ saturation (%) | 97.4 ± 2.8 | 96.0 ± 7.6 | 0.001 | 97.3 ± 3.5 | 96.8 ± 5.4 | 0.297 |
| Lactate (mg/dL) | 17.7 ± 14.4 | 34.6 ± 32.7 | <0.001 | 17.6 ±14.7 | 33.4 ± 31.0 | <0.001 |
| Fraction of Inspired Oxygen (%) | 42.9 ± 15.4 | 52.1 ± 22.0 | <0.001 | 44.7 ± 14.4 | 50.9 ± 20.7 | <0.001 |
